# Identifying the Factors Associated With Spatial Clustering of Incident HIV Infection Cases in High-Prevalence Regions: Quantitative Geospatial Study

**DOI:** 10.2196/75291

**Published:** 2025-09-16

**Authors:** Qiyu Zhu, Chunnong Jike, Chengdong Xu, Shu Liang, Gang Yu, Dan Yuan, Hong Mai, Yiping Li, Lin Xiao, Ju Wang, Hong Yang, Fengshun Yuan, Jing Hong, Muga Mao, Maogang Shen, Jing Liu, Lin He, Yuehua Wang, Huanyi Cheng, Peng Guan, Yan Jiang, Mengjie Han, Cong Jin, Zhongfu Liu

**Affiliations:** 1 National Key Laboratory of Intelligent Tracking and Forecasting for Infectious Diseases National Center for AIDS/STD Control and Prevention Chinese Center for Disease Control and Prevention Beijing China; 2 China Medical University Shenyang China; 3 Liangshan Prefecture Center for Disease Control and Prevention Xichang China; 4 Institute of Geographic Sciences and Natural Resources Research, Chinese Academy of Sciences Beijing China; 5 Sichuan Provincial Center for Disease Control and Prevention Chengdu China; 6 The First People's Hospital of Yi Autonomous Prefecture of Liangshan Xichang China

**Keywords:** HIV, disease transmission, spatial analysis, geographic mapping, public health

## Abstract

**Background:**

Incident HIV infection is a critical indicator of an ongoing epidemic, particularly in high-burden regions such as Liangshan Yi Autonomous Prefecture in China, where HIV prevalence exceeds 1% in 4 key counties (Butuo, Zhaojue, Meigu, and Yuexi). Identifying spatial clusters and drivers of recent infections is essential for implementing targeted interventions. Despite advancements in geospatial analyses of HIV prevalence, studies identifying drivers of incident HIV clustering remain limited, especially in low-resource settings.

**Objective:**

This study aims to identify spatial clusters of recent HIV infections and investigate potential driving factors in 4 key counties of the Liangshan Yi Autonomous Prefecture to inform targeted intervention strategies.

**Methods:**

From November 2017 to June 2018, we identified 246 (4.42%) recent HIV infection cases from 5555 newly diagnosed cases through expanded testing of the whole population in 4 key counties of Liangshan Yi Autonomous Prefecture. Recent infection cases were confirmed using limiting antigen avidity enzyme immunoassays or documented seroconversion within 6 months. The spatial distribution of incident HIV infection cases was analyzed using kernel density. Potential drivers, including population density, HIV prevalence, elevation, nighttime light index, urban proximity, and antiretroviral therapy (ART) coverage, were analyzed. The spatial lag regression model was used to identify factors associated with clustering of recent infection cases. The Geodetector *q*-statistic was used to quantify nonlinear interactive effects among these factors.

**Results:**

Significant spatial autocorrelation was observed in the distribution of recent HIV cases (Moran *I*=0.11; *P*<.01). Six spatial clusters were identified, and all were located near urban centers or major roads. Furthermore, 5 factors were identified by the spatial lag regression model as being significantly correlated with the clustering of recent HIV infection cases, including population density (β=0.59; *P*<.001), HIV prevalence (β=0.02; *P*<.001), distance to local urban area (β=–3.10; *P*=.01), SD of elevation (β=–0.15; *P*=.02), and ART coverage rate (β=183.80; *P*<.01). Geodetector analysis revealed strong interactive effects among these 5 factors, with population density and HIV prevalence exhibiting the largest interactive effect (*q*=0.69).

**Conclusions:**

This study reveals that besides HIV prevalence, urbanization-related factors (population density and proximity to urban area) and transportation accessibility drive incident HIV clustering in Liangshan Yi Autonomous Prefecture. Paradoxically, higher ART coverage was associated with increased transmission, suggesting the need for integrated prevention strategies beyond ART expansion. Furthermore, the township-level geospatial approach provides a valuable model for pinpointing transmission hot spots and tailoring interventions in high-burden regions globally.

## Introduction

### Background

Incident HIV infection is a direct indicator of an ongoing epidemic. Collecting detailed information on new infection cases has been advocated by the World Health Organization and the Joint United Nations Programme on HIV/AIDS, especially in high-burden areas, to inform trends of HIV transmission. Several laboratory-based assays, such as limiting antigen avidity enzyme immunoassay, have been developed to identify recent HIV infection from newly diagnosed cases and have been proven valid with good credibility [1,2]. The applications of assays for recent HIV infection include providing national estimates of incidence, monitoring HIV incidence in key or sentinel populations, assessing the impact of interventions, and identifying clusters and populations of current epidemics [3-7].

An emerging application of recent HIV infection testing is its integration with geospatial analysis to detect geographic clusters of new transmission, thereby supporting targeted interventions. In Malawi, 3 hot spots of recent infection cases were identified with facility-based HIV testing data [8]. The cluster also indicated service delivery gaps and required an immediate public health response [9]. In several provinces of China, recent infection testing was performed among newly diagnosed HIV cases, and spatial autocorrelation and spatial scan statistics were used to detect hot spots [10-13]. Moreover, in a border prefecture of China, a Bayesian spatiotemporal model was used to estimate the dynamic spatial relative risk of recent infections [14]. Most existing studies focused on describing the spatial distribution, estimating the relative risks, and detecting the hot spots of recent infection cases. However, studies specifically analyzing the potential driving factors of the spatial clustering of incident HIV infection cases remain limited, particularly in low- and middle-income settings and regions with high HIV burden.

Liangshan Yi Autonomous Prefecture, located in Sichuan province in the southwest of China, is one region that has been severely affected by HIV [15,16]. The HIV infection rate among residents in Liangshan Yi Autonomous Prefecture was 0.84% in 2019, with the HIV infection rate in 4 key counties of Butuo, Zhaojue, Meigu, and Yuexi in Liangshan Yi Autonomous Prefecture exceeding 1% [17]. The key to controlling the HIV epidemic in this region is to reveal critical driving factors affecting HIV transmission and implement targeted interventions.

### This Study

In this study, we tried to focus on 246 recent HIV infection cases identified from 5555 newly diagnosed HIV cases through an expanded HIV testing from November 2017 to June 2018 in the general population in the 4 key counties. Furthermore, we retrospectively conducted multiple geospatial analyses to identify clusters and high-risk areas of recent HIV infection, aiming at revealing potential factors driving the clustering of incident HIV infection cases in this region and consequently informing targeted interventions to effectively curb the local epidemic.

## Methods

### Study Scheme

The newly diagnosed HIV cases in this study were identified through expanded HIV testing in residents of the 4 key counties of Butuo, Zhaojue, Yuexi, and Meigu in Liangshan Yi Autonomous Prefecture from November 2017 to June 2018; the testing was incorporated into a physical examination provided as a public health service covering more than 95% of the residents. Participating individuals were informed of HIV testing and could choose to reject the test without impacting other parts of the physical examination service. The individuals signed a physical examination form to approve all testing. For individuals who agreed to HIV testing, a rapid test for detecting HIV specific IgM and IgG (Wondfo) antibodies was performed. Individuals with positive screening results were asked to undergo a confirmatory immunoblot test (MP Biomedicals), and the individuals with positive immunoblot test results were registered in the national HIV database.

### Identification of Recent HIV Infection Cases

Among newly diagnosed HIV cases, recent HIV infection cases were identified in 2 ways. Most (196/246, 79.7%) of the recent HIV infection cases were identified by a limiting antigen avidity enzyme immunoassay (Beijing Kinghawk Pharmaceutical Co, Ltd). According to the manufacturer’s instructions, the mean duration of recent infection of this assay was 130 days. Recent HIV infection cases were also identified through HIV testing history of newly diagnosed HIV cases as having a known negative HIV testing result within 6 months before a positive HIV testing result during the survey period.

### Sources of Data

**Figure 1:**
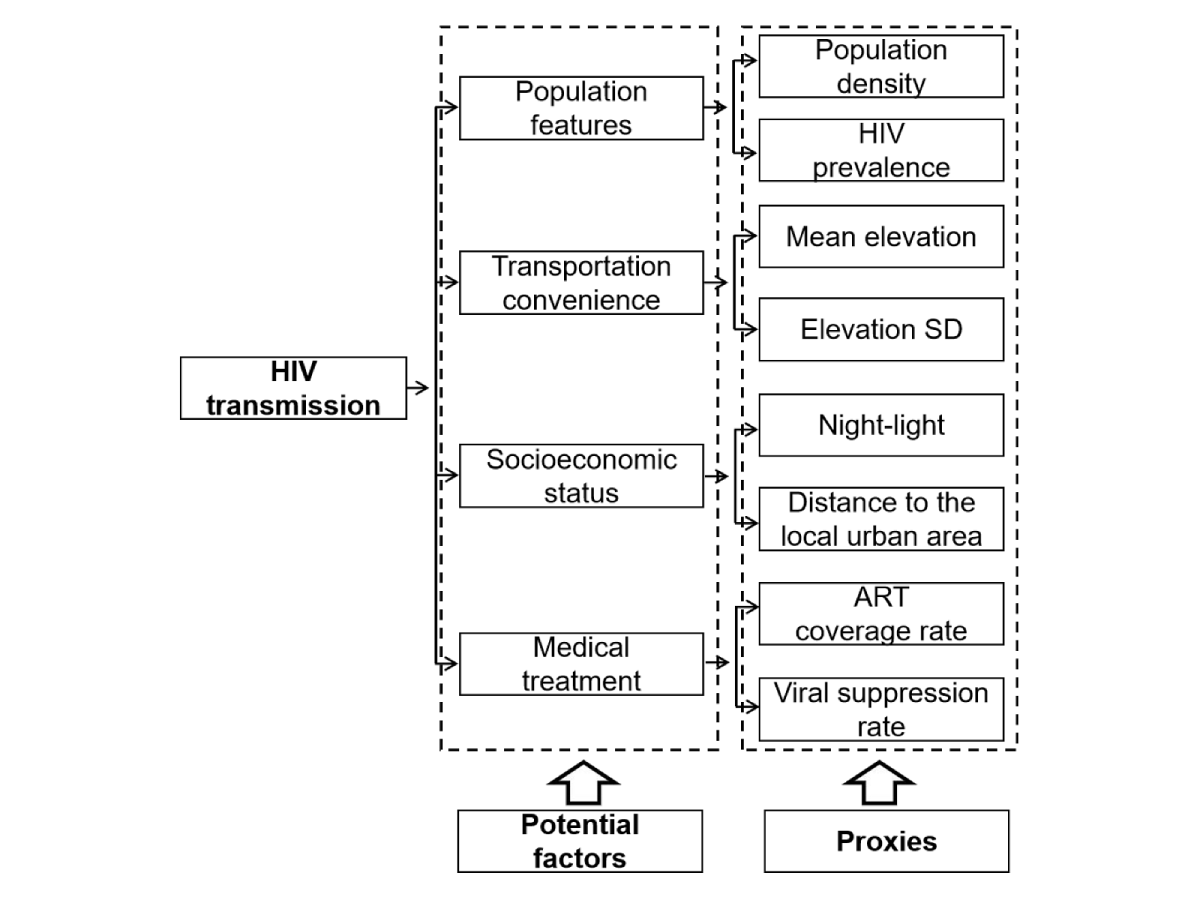
Potential driving factors and their proxy factors of HIV transmission. Population features, transportation convenience, socioeconomic status, and medical treatment are 4 potential driving factors of HIV transmission. Population density, HIV prevalence, mean elevation, elevation SD, night-light, distance to local urban center, antiretroviral therapy (ART) coverage rate, and viral suppression rate serve as proxy variables of potential driving factors.

In this study, we analyzed potential factors related to the spatial clustering of recent infection cases. On the basis of previous studies [18,19] and local contextual relevance, we categorized these factors into four aspects: (1) population features, (2) transportation convenience, (3) socioeconomic status, and (4) medical treatment (Figure 1).

The first aspect was population features. Population density (persons/km²) and HIV prevalence were included to reflect the level of population aggregation and the underlying burden of infection, respectively. Information for calculating population density in the township was obtained from the local statistics bureau. HIV prevalence data were provided by the prefecture center for disease control and prevention.

The second aspect was transportation convenience. Previous studies have shown that transportation accessibility can influence HIV transmission and outcomes [20,21]. Given the mountainous terrain in Liangshan Yi Autonomous Prefecture, variations in elevation may lead to disparities in transportation infrastructure and accessibility. Geographic information on elevation was obtained from the National Catalogue Service for Geographic Information. The mean and SD of elevation were calculated using ArcGIS software (Esri) as proxy variables of transportation convenience.

The third aspect was socioeconomic status. Due to data unavailability, we included the nighttime light index and distance to the local urban area as proxies for socioeconomic status. Nighttime light data were sourced from a platform developed by the Chinese Academy of Sciences, and township-level distances to local urban centers were calculated using ArcGIS software.

The fourth aspect was medical treatment. HIV antiretroviral therapy (ART) coverage and viral suppression are 2 important indicators of HIV treatment. These indicators reflect the availability and effectiveness of HIV treatment services. Data on ART coverage and viral suppression rates were obtained from the local ART quality control center.

Furthermore, demographic and location information of recent HIV infection cases were collected from the national HIV/ AIDS database. Recent HIV infection cases were mapped using village midpoint coordinates extracted from resident address information.

### Geographic and Statistical Analyses

All geographic maps were created using ArcGIS (version 10.6). General Moran *I* index was calculated using ArcGIS to determine the spatial autocorrelation of township-level aggregated number of recent infection cases. Kernel density estimation was performed with ArcGIS to construct a smooth surface for the geographic distribution of recent HIV infection cases. Ordinary least squares regression (OLSR) and spatial lag regression (SLR) were performed using GeoDa (version 1.12.1.139) software to determine the linear correlation between the kernel density of recent HIV infection cases and potential factors. Geodetector *q*-statistic was used to investigate the interactive effect (linear or nonlinear) of a single or every 2 of the proxies on the kernel density of recent HIV infection cases. In general Moran *I* index, OLSR, SLR, and Geodetector *q*-statistic, the statistical unit was township, which means that township-level data were used for analysis. Statistical analyses were performed using SPSS Statistics (version 23.0; IBM Corp) statistical analysis software package. Frequencies of variables were compared using the Fisher exact chi-square test. Considering heterosexual transmission has become the main route of HIV infection, the demographic characteristics between different genders were compared.

### Kernel Density Estimation

Kernel density estimation (equation 1) is a process of inferring the probability density at each location, that is, constructing the probability density surface, based on the locations of residents (the points), and it can visualize the density of recent cases of infection. This method has been widely used to construct a smooth surface for the geographic distribution of HIV cases [22,23]. In this study, kernel density estimation was performed using geographic information of recent cases of HIV infection within the study area. The analysis was conducted in ArcGIS software with default parameters, and the study area boundary was used to define the extent of the analysis. The kernel density was calculated as follows [24,25]:



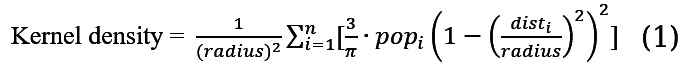



For *dist_i_*<*radius*

where *dist_i_* specifies the distance between the point of the recent HIV infection case and location (x, y), *n* represents the total number of recent HIV infection cases, *radius* is the kernel bandwidth, and *pop_i_* is the number of recent HIV infection cases in point . To enable township-level analysis, the average kernel density value within each township was further calculated using ArcGIS.

### General Moran I Index

The general Moran *I* index (equation 2) was calculated using ArcGIS software to determine the spatial autocorrelation of township-level aggregated numbers and kernel density of recent infection cases. The general Moran *I* index was calculated as follows [26]:



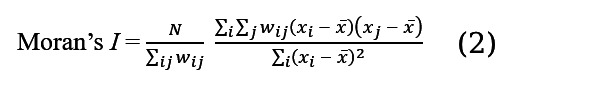



where *N* represents the number of townships in the study area (*N*=151); *w*_ij_ denotes the spatial weight between townships *i* and *j*; *x_i_* and *x_j_* are the values of kernel density in the township *i* and *j*, respectively; and specifies the mean kernel density in the study area. The value of the general Moran *I* index ranged between –1 and 1 (positive autocorrelation: Moran *I*>0; negative autocorrelation: Moran *I*<0).

### Linear Regression Model

We used both the OLSR model (equation 3) and the SLR model (equation 4) to identify factors associated with the clustering of recent infection cases. These 2 models assumed linear relationships between the dependent variable and explanatory variables. The SLR model further incorporated spatial dependence into the regression model with a spatially lagged dependent variable, which accounted for the fact that the dependent variable (*y*) may be spatially autocorrelated [27]. The fitness of these 2 models was compared to determine which model was most appropriate for analysis. OLSR and SLR were denoted as follows:

y = Xβ + ε **(3)**

y = ρWy + Xβ + μ **(4)**

In both equations, *y* is a vector of kernel density of recent infection cases in each township (N=151), *X* is a vector of explanatory variables, and β is the regression coefficient of the explanatory variable. In the OLSR model (equation 3), *ε* represents the random error. In the SLR model (equation 4), ρ is the coefficient of the spatial lag parameter and represents the level of autocorrelation in the lag term, *W_y_* is a vector of spatial weights, and *μ* specifies the nonspatial random error. Queen contiguity was used to assign spatial weights to townships (nonzero weights for townships that share a common edge or vertex and zero weights to the others).

### Geodetector

Geodetector (Institute of Geographic Sciences and Natural Resources Research, Chinese Academy of Sciences), a statistical tool, can measure the spatial stratified heterogeneity and quantify the influencing power of a single or every 2 of the proxies included. Geodetector assumes that explanatory variable and dependent variable would be coupled (linear or nonlinear) in strata if they are correlated, and the nonlinear assumption is its advantage. Thus, we used Geodetector to quantify the influencing power of a single or every 2 proxies with the *q*-statistic value. The *q*-statistic value was expressed as follows [28]:



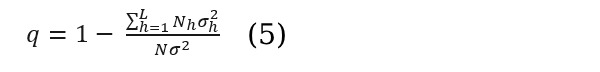



where *h* (*h*=1,2,...*L*) is the stratification of a single factor (*X*) or every 2 interacting factors, *N_h_* and *N* represent the number of townships in the stratum *h* (h=1,2,...,*L*) and the entire area separately (the stratification was calculated by natural break), and and denote the variances of the kernel density in the stratum *h* and the whole study area, respectively. The *q*-statistic value ranged between 0 and 1, and a larger value indicated higher influencing power.

### Ethical Considerations

This study, which involved analyzing the data on recent HIV infection cases identified through expanded HIV testing in Liangshan Yi Autonomous Prefecture, was approved by the institutional review board of the National Center for AIDS/STD Control and Prevention, Chinese Center for Disease Control and Prevention. This expanded HIV testing was incorporated into the local physical examination. All participants signed informed consent forms at the time of their examinations. The physical examination was free, and no financial compensation was provided to participants. No personally identifiable information was present in the data analyzed for this study.

## Results

### Demographic Characteristics of Recent HIV Infection Cases

A total of 246 (4.4%) individuals who were recently infected with HIV were identified from 5555 newly diagnosed cases in 4 key counties in Liangshan Yi Autonomous Prefecture. A total of 217 (88.2%) of them provided complete demographic information and were included in the analysis of demographic characteristics. Among the 217 individuals who were newly infected (Table 1), 92 (42.4%) were male and 125 (57.6%) were female. In both male and female, individuals aged between 30 and 44 years, those who were married, and those who had not attended school occupied the largest proportion. A significant difference was seen in the transmission modes between different genders (*P*<.001). A large proportion of individuals who were recently infected acquired HIV through heterosexual transmission, and this number was 87.2% (109/125) and 62% (57/92) in female individuals and male individuals, respectively.

**Table 1:** Demographic characteristics of individuals who were recently infected with HIV in 4 key counties of Liangshan Yi Autonomous Prefecture, China, from November 2017 to June 2018.

Demographic characteristic			Total (N=217), n (%)		Male (n=92), n (%)		Female (n=125), n (%)		*P* value^a^
**Age (y)**									.33
	1.5-14	15 (6.9)		5 (5.4)		10 (8)			
	15-29	69 (31.8)		24 (26.1)		45 (36)			
	30-44	103 (47.5)		49 (53.3)		54 (43.2)			
	≥45	30 (13.8)		14 (15.2)		16 (12.8)			
**Marital status**									.45
	Single	56 (25.8)		25 (27.2)		31 (24.8)			
	Married	135 (62.2)		53 (57.6)		82 (65.6)			
	Divorced or widowed	23 (10.6)		13 (14.1)		10 (8)			
	Unknown	3 (1.4)		1 (1.1)		2 (1.6)			
**Educational attainment**									.15
	No schooling	143 (65.9)		54 (58.7)		89 (71.2)			
	Primary school	66 (30.4)		34 (37)		32 (25.6)			
	Middle school and above	8 (3.7)		4 (4.3)		4 (3.2)			
**Infection routes**									<.001
	Heterosexual contact	166 (76.5)		57 (62)		109 (87.2)			
	Drug injection	36 (16.6)		30 (32.6)		6 (4.8)			
	Others	15 (6.9)		5 (5.4)		10 (8)			

^a^Statistical significance was assessed between male and female individuals by the Fisher exact test.

### Spatial Distribution and Clusters of Recent HIV Infection Cases

**Figure 2:**
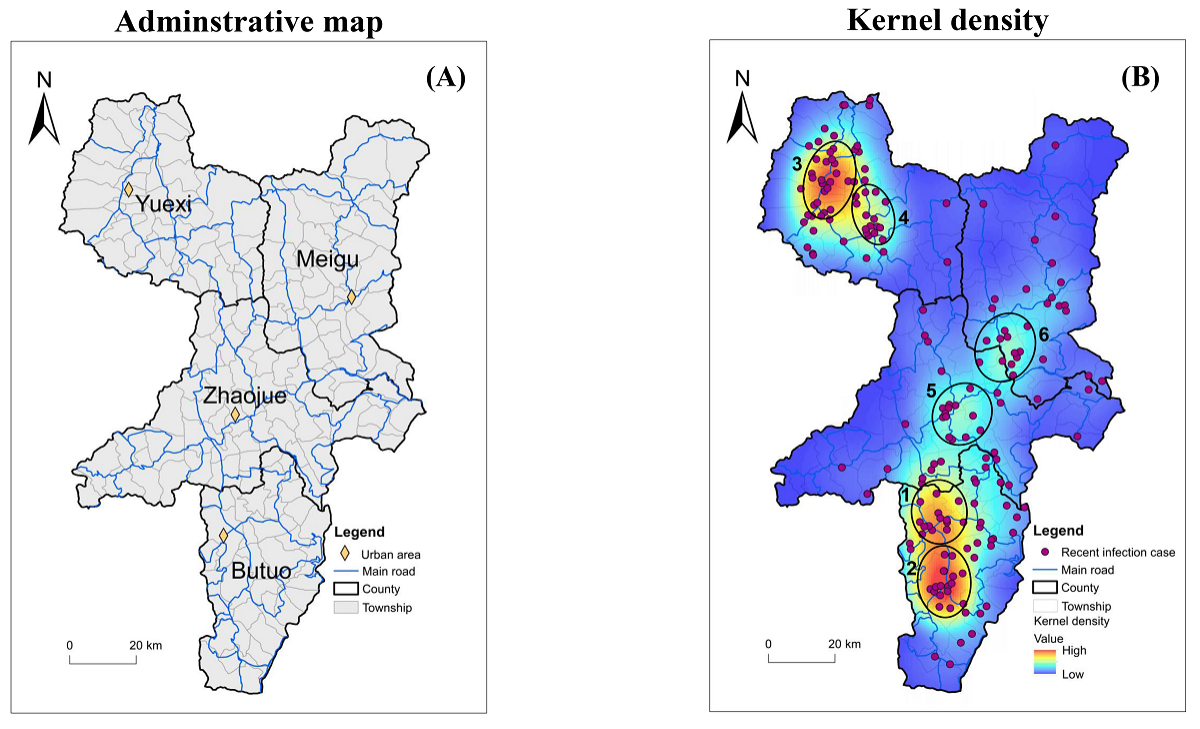
Spatial distribution and high-risk areas of recent HIV infection cases in 4 key counties of Liangshan Yi Autonomous Prefecture, China, from November 2017 to June 2018. (A) Administrative map of the 4 key counties. Blue lines indicate main traffic roads, and yellow diamonds indicate the local urban areas of each county. (B) The spatial distribution of recent HIV infection cases is shown using a kernel density map. The color-code shows the density of recent HIV infection cases per square map unit. The black circles show clusters of recent HIV infection cases.

The general Moran *I* value of township-level kernel density of recent HIV infection cases was 0.11 (*P*<.01), which indicated significant positive spatial autocorrelation. To investigate spatial distribution features of recent HIV infection cases in the 4 neighboring counties (Figure 2A), the kernel density of recent HIV cases was calculated and mapped. We found 6 main clusters of recent HIV cases, and all 6 clusters were located at the intersection of main traffic roads (Figure 2B). Except for cluster 6 at the border of Zhaojue and Meigu, which is a transportation portal between Zhaojue and Meigu, other clusters were located around urban areas in the county. Our spatial distribution analyses also indicated spatial variation of recent HIV infection.

### Factors Associated With the Clustering of Recent HIV Infection Cases

The general Moran *I* value of township-level kernel density of recent infection cases was 0.76 (*P*<.001), which indicated significant positive spatial autocorrelation. Therefore, we further used the spatial regression analysis, SLR, which accounts for the fact that the dependent variable (*y*) may be spatially autocorrelated, to perform quantitative analysis and identify factors significantly associated with clustering of recent HIV infection cases and compare it with a nonspatial model, OLSR.

**Figure 3:**
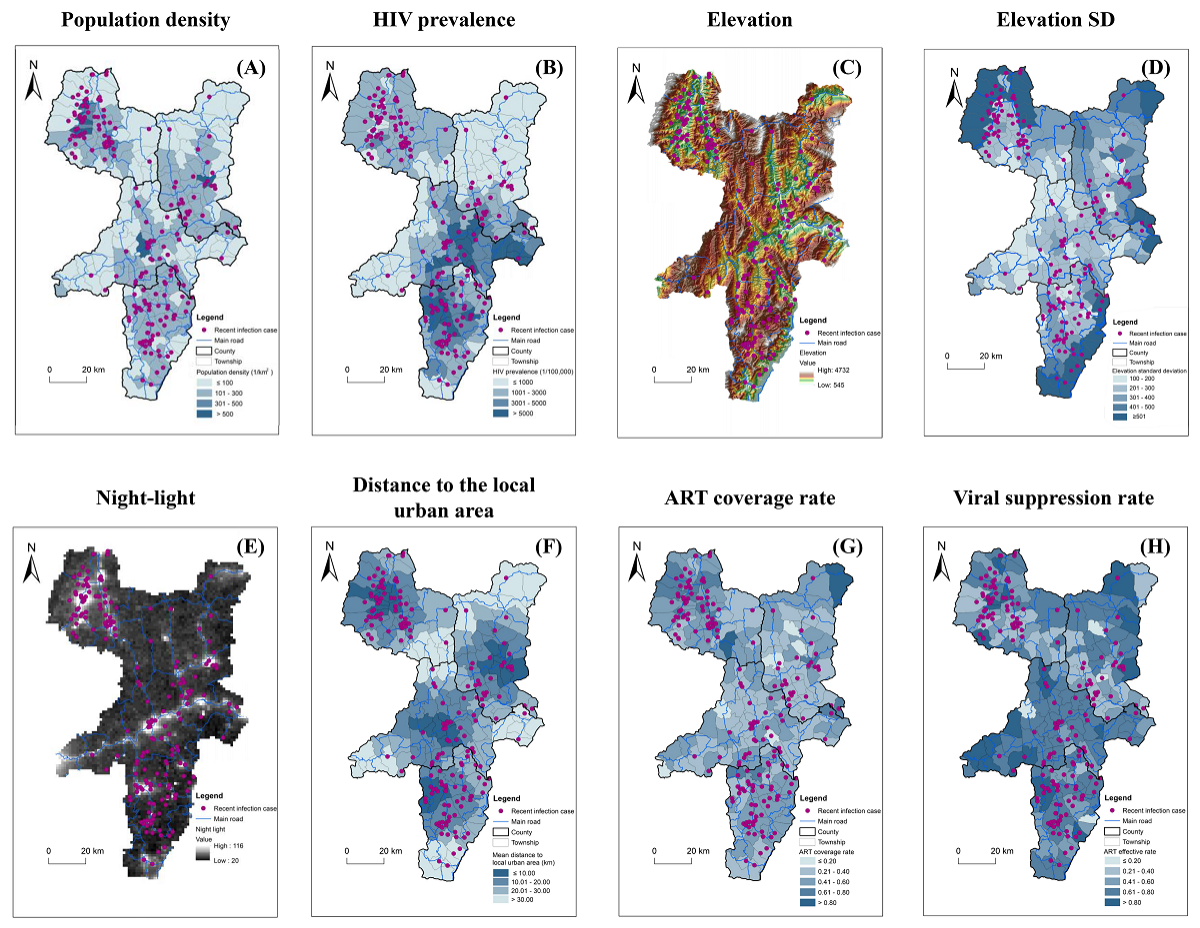
Maps of 8 potential factors contributing to the clustering of recent HIV infection cases in 4 key counties of the Liangshan Yi Autonomous Prefecture, China, from November 2017 to June 2018. (A) Map of population density at the township level. The color intensity shows the number of individuals per square km. (B) Map of HIV prevalence at the township level. The color intensity shows the level of HIV prevalence. (C) Elevation map. The color-code shows elevation. (D) Elevation SD map. The color-code shows elevation SD. (E) Night-light map. The color-code shows the nighttime light index. (F) Map of distance to local urban center at the township level. The color intensity shows the distance range. (G) Map of antiretroviral therapy (ART) coverage rate at the township level. The color intensity shows the level of ART coverage rate. (H) Map of the viral suppression rate at the township level. The color intensity shows the level of the viral suppression rate.

We investigated the factors that might impact the clustering of recent HIV infection cases while considering the unique geographic, demographic, and HIV epidemic features in the area. Population density and HIV prevalence rate of the township were incorporated into the analysis, and we found that all clusters were located in areas with high population density and high HIV prevalence (Figures 3A and 3B). Because the 4 key counties are located in a mountainous area, the altitude was taken into account. The topographical map showed that, except for cluster 2 in Butuo and cluster 4 in Yuexi, all other clusters were located in areas at a lower altitude (Figure 3C). Elevation SD indicates the steepness of local terrain, which can impact the convenience of transportation to a certain extent (Figure 3D). Nighttime light index was used to represent the general economic status at the township level. Because the 4 key counties are lower-income areas, most areas are dark. We found that, except for cluster 2 in Butuo, all other clusters were located in areas with bright night-light, indicating comparatively better economic status (Figure 3E). The distance to the local urban areas of the counties was also mapped, and it showed that all clusters were located within 20 km of the urban areas (Figure 3F). The impact of ART coverage rate and viral suppression rate of individuals infected with HIV before the survey was also examined. The results showed that all clusters were mainly located in areas with comparatively higher ART coverage rates (Figure 3G). Clear patterns related to the distribution relationship between the clusters and the viral suppression rate were not noted (Figure 3H).

In addition to visually mapping potential factors, we used a nonspatial OLSR model and an SLR model to perform quantitative analysis and identify proxies significantly associated with clustering of recent HIV infection cases. Geographic kernel density of recent HIV infection cases was used to represent clustering intensity, and the correlation between kernel density of recent infection cases and proxies, including population density, HIV prevalence, mean elevation, elevation SD, night-light, distance to local urban area, ART coverage rate, and viral suppression rate, was analyzed.

In comparison to the nonspatial OLSR analysis, the SLR model fit better, and there was a significant spatial autocorrelation in the township-level kernel density of recent infection cases (ρ=0.72; *P*<.001; Table 2). In the SLR model, 85% of the spatial variations in kernel density of recent infection cases can be explained by the proxies included (higher than 62% in the nonspatial OLSR model). In the SLR model, at the township level, population density, HIV prevalence, and ART coverage rate had a positive correlation with kernel density of recent infection cases, while elevation SD and distance to local urban areas had a negative correlation.

**Table 2:** Results from nonspatial and spatial linear models, namely ordinary least squares regression (OLSR) and spatial lag regression (SLR), and proxy factors associated with clustering of recent HIV infection cases in 4 key counties of Liangshan Yi Autonomous Prefecture, China, from November 2017 to June 2018.

Proxy factors	OLSR^a^		SLR^b^	
	β coefficient	*P* value	β coefficient	*P* value
Population density	0.91	<.001	0.59	<.001
HIV prevalence	0.05	<.001	0.02	<.001
Mean elevation	0.03	.58	–0.03	.45
Elevation SD	0.09	.40	–0.15	.02
Night-light	–7.61	.23	–2.93	.45
Distance to the local county urban area	–11.11	<.001	–3.10	.01
ART^c^ coverage rate	491.52	<.001	183.80	<.01
Viral suppression rate	11.73	.88	19.73	.68

^a^*R*^2^=0.62; *P*<.001; Akaike information criterion=2022.78.

^b^*R*^2^=0.85; ρ=0.72 (represents the level of autocorrelation in the lag term); Akaike information criterion=1911.81; *P*<.001.

^c^ART: antiretroviral therapy.

**Figure 4:**
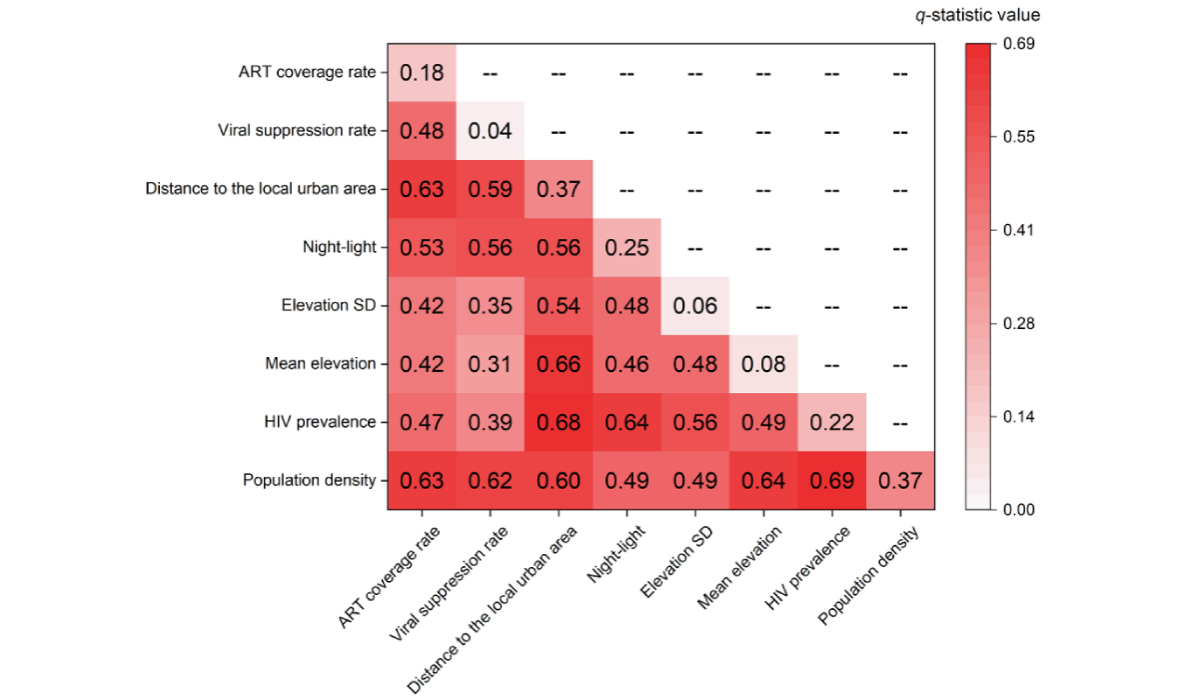
Interactive effects of 8 potential factors on clustering of recent HIV infection cases in 4 key counties of Liangshan Yi Autonomous Prefecture, China, from November 2017 to June 2018. The color intensity at the intersection of 2 factors shows the extent to which the 2 factors interactively contribute to clustering of recent HIV infection cases. The regions are color-coded from pale red to dark red, and dark red indicates a high q-statistic value. ART: antiretroviral therapy.

To further elucidate the nonlinear influencing power of a single and every two proxies on the clustering of recent HIV infection cases, we performed Geodetector analysis. Our results revealed that population density and HIV prevalence showed the highest interactive influence on clustering of recent HIV infection cases, reaching a *q*-statistic value of 0.69 (Figure 4). Furthermore, for every 2 of the 5 proxies, including HIV prevalence, population density, distance to local urban area, SD of elevation, and ART coverage rate, a strong interactive effect was observed through Geodetector analysis (Figure 4). Regarding the influencing power of the single proxy factor, population density and distance to the local urban area had the highest *q*-statistic value of 0.37 (Figure 4).

## Discussion

### Principal Findings

Many potential factors could impact local HIV transmission, such as local HIV prevalence, socioeconomic status, transportation convenience, access to HIV-related health care, local culture, etc [19,29-32]. On the basis of the data available at the township level, we categorized the potential driving factors into 4 aspects, which were population features, transportation convenience, socioeconomic status, and access to HIV care. For every potential driving factor that we proposed to study, we tried to use the most appropriate proxy variables for the analyses. Due to a lack of data at the township level for other potential factors, for example, residents’ demographic information, access to education, harm reduction, gross domestic product, urbanization situation, and preexposure prophylaxis awareness, these were not included in this study. We also did not include some potential factors that are difficult to quantify, such as attitudes toward casual sex behavior in the Yi ethnic group [19]. However, it is compelling that the factors that we analyzed could explain the clustering of recent HIV infection cases for a high proportion of 85% in the SLR analysis (Table 2). Therefore, these potential factors included in our regression analyses showed a strong explanatory effect on the clustering of recent HIV infection cases.

Our novel geospatial analyses focusing on incident HIV infection allowed us to capture the underlying drivers of the ongoing HIV epidemic. In our study, the geographic distribution of recent HIV infection cases at the township-level has a significant spatial autocorrelation, and the SLR model fitted better than the nonspatial OLSR model, which did not consider spatial features of the area. The SLR model identified 5 factors—population density, HIV prevalence, elevation SD, the distance to the local urban area, and ART coverage rate. In the nonlinear Geodetector analysis, population density and the distance to local urban areas independently contributed to the highest *q*-statistic value of 0.37, and they were the 2 main parameters used to define urbanization [33]. Compared to the nonspatial OLSR model, the SLR model further identified significant factors such as elevation SD, which indicates the steepness of the road, and was used to represent the convenience of transportation. Therefore, our results suggest that areas with higher levels of urbanization and more convenient transportation in the 4 key counties are more inclined to have spatial clustering of incident HIV infection cases. A previous study using spatial clustering analysis of newly diagnosed cases in Mississippi, United States, also showed that urban residence was an important risk factor for HIV acquisition [30] and postulated that HIV spread more rapidly in highly populated urban areas with dense sex work networks [34]. Another study in Lilongwe, Malawi, indicated that due to the proximity to transportation and commercial networks, urbanization may increase the rates of HIV transmission and put women at a higher risk than men [32]. According to a quantitative study in Africa, a 10% increase in urbanization could be associated with a 3.6% increase in adult HIV prevalence [35]. However, while all previous studies used HIV prevalence data for their analysis, our study used HIV incident infection data to reveal the link between urbanization and HIV transmission, an indicator that could serve as a more direct reflection of an ongoing epidemic. Our findings suggest that local governments in areas undergoing urban development should allocate resources toward education on healthy sex behavior to the public and provide related preventive measures to reduce the potential transmission risk of HIV infection.

In our study, the SLR also revealed HIV prevalence and ART coverage rate as significant factors. Meanwhile, the Geodetector analysis showed that both factors had a high interactive effect with population density and distance to the local urban area. Thus, it suggests that these 2 factors may contribute to HIV transmission in Liangshan in a more complex, nonlinear way. It is not surprising that HIV prevalence had a positive relation with HIV transmission. However, it is intriguing that our study found a positive relation between ART coverage and HIV transmission because it has been acknowledged by many studies that expanded ART coverage is associated with decreased HIV transmission [36]. It has been debated whether the preventive effects of ART at an individual level, as shown in the HIV Prevention Trials Network 052 trial in heterosexual couples with HIV serodiscordance, can be replicated at a population level and whether outcomes from mathematical models can be replicated in the real world [37-39]. Early infection, consistent risk behaviors, adherence to therapy, and drug resistance were factors suggested to compromise the preventive effect of expanded antiretroviral coverage [38,39]. In the 4 key counties, there could be higher ART coverage rate in urban areas with better health care services; however, including the context of an extensive sexual network in urban areas and considering the general environment of insufficient detection of HIV infection and poor drug adherence in this area [40], relying solely on expansion of ART may not result in effective reduction in the transmission and incidence of new infections. In addition, the level of pretreatment drug resistance has been moderate in these 4 counties [41]. Therefore, combination prevention strategies, including universal testing, expansion of treatment, increased drug adherence, and other combined targeted measures, are needed to effectively avert the epidemic in the Liangshan area.

### Limitations

There are several limitations in our study. The analysis of incident infection cases is generally impacted by the individuals who are newly diagnosed, identified through the local testing program. In this study, the expanded HIV testing covered more than 95% of the residents; therefore, this impact was minimized. However, approximately 5% of the residents were not included in this study due to out-migration for work and population movement, which might still lead to a certain level of bias. It is also noteworthy that the HIV recency assay has a false recent rate (FRR) such that some cases of long-term infection could be misclassified as recent infection. In this study, we excluded HIV cases that received ART and determined the FRR as 1.48% in the 4 key counties. Considering the low FRR, our main findings should not be affected. Furthermore, in modeling the spatial distribution of recent HIV infection cases, kernel density was calculated to reflect the clustering of recent HIV infection cases to address the limited number of recent infections. While kernel density is not a standard epidemiological indicator and its use may affect model interpretation, it serves as a useful preliminary tool for identifying potential clustering patterns. Future studies with larger datasets and more stable incidence rates are suggested to refine this approach.

### Conclusions

Geospatial studies explicitly focused on new HIV infection cases are rare [11]. To the best of our knowledge, our study is the first to use spatial regression analysis to reveal factors associated with clustering of incident HIV infection cases. After revealing 6 clusters of incident HIV infection, we further identified population density, HIV prevalence, elevation SD, distance to local urban area, and ART coverage rate as 5 factors impacting current HIV transmission in the 4 key counties that were severely impacted by the HIV epidemic. Our novel geospatial study at the township level provides a new tool for zooming into a local area and examining unique factors in detail. We hope our analytical tools will be useful in identifying high-risk regions and populations in other areas of high HIV prevalence and thus inform more targeted public health responses, which can be used to avert epidemics.

## Abbreviations

ART: antiretroviral therapy
FRR: false recent rate
OLSR: ordinary least squares regression
SLR: spatial lag regression

## Data Availability

The datasets generated or analyzed during this study are available from the corresponding author on reasonable request.
